# Supplementary material for: Dopamine-Depleted Dopamine Transporter Knockout (DDD) Mice: Dyskinesia with L-DOPA and Dopamine D1 Agonists
Source: Biomolecules. 2023 Nov 17;13(11):1658. doi: 10.3390/biom13111658 (PMC10669682; doi:10.3390/biom13111658)
Supplement: Supplementary file 1 [file biomolecules-13-01658-s001.zip › Table S1.pdf]

Supplementary Table S1. Effects of D1R compounds on oral stereotypies in DDD mice[a].

	Oral Stereotypy[b]	
Drug[c,d]	OF[d,e,f]	CM[d,e,f]
Vehicle	0.5 ±0.21	0.1 ±0.09
5 SKF	2.4 ±0.48[g]	1.8 ±0.27[ggg]
5 MLM	1.9 ±0.43	3.1 ±0.60[gg]
10 MLM	1.9 ±0.34[g]	3.0 ±0.59[gg]

[a]DDD mice were treated with 125 mg/kg AMPT plus 6/12.5 mg/kg L-DOPA/ Benz and tested with compounds (Figure 1A).
[b]Behaviors were scored beginning at 5-min after drug administration at 10-min intervals over 85 min.
[c]Doses of drugs (in order): 5 mg/kg SKF81297, 5 mg/kg MLM55-38, and 10 mg/kg MLM55-38.
[d,]Abbreviations: OF, open field; CM, circular maze; SKF, SKF81297; MLM, MLM55-38.
[e]ANOVA: OF [drug: $F(3,39)=7.077$, $P=0.014$], Welch ANOVA CM [drug: $F(3,16.473)=21.686$, $P<0.001$].
[f]Results presented as means ±SEMs; N=10-11 mice/group (OF) and N=9-11 mice/group (CM).
[g]$P<0.05$, [gg]$P<0.01$, [ggg]$P<0.001$, vehicle *vs*. drug groups within test context.
