# Supplementary material for: Dopamine-Depleted Dopamine Transporter Knockout (DDD) Mice: Dyskinesia with L-DOPA and Dopamine D1 Agonists
Source: Biomolecules. 2023 Nov 17;13(11):1658. doi: 10.3390/biom13111658 (PMC10669682; doi:10.3390/biom13111658)
Supplement: Supplementary file 1 [file biomolecules-13-01658-s001.zip › Table S2.pdf]

Supplementary Table S2. Effects of D1R compounds on DDD mice without L-DOPA/Benz[a].

	Vehicle[b,c]	5 mg/kg SKF[b,c]	5 mg/kg MLM[b,c]	10 mg/kg MLM[b,c]
Distance Traveled[d,e]	31.5 ±13.86	589.9 ±102.04[fff]	163.3 ±27.33[ff]	273.8 ±54.10[ff]
Vertical Activity Counts[d]	28.2 ±25.32	24.5 ±9.97	28.1 ±24.24	25.0 ±20.92
Supported Rearing[d]	0.0 ±0.00	0.2 ±0.17	0.1 ±0.07	0.1 ±0.07
Climbing[d]	0.0 ±0.00	0.0 ±0.00	0.0 ±0.00	0.0 ±0.00
Oral Stereotypy[d]	0.0 ±0.00	0.0 ±0.00	0.1 ±0.07	0.1 ±0.07

[a]The DDD mice had been sensitized to 6/12.5 mg/kg L-DOPA/Benz (with AMPT) and tested initially with the vehicle and D1R agonists (Figure 1). They were withdrawn from all treatments for 1 wk prior to testing responses (day 27) to the D1R agonists in the absence of L-DOPA/Benz but in the presence of AMPT.

[b]Results presented as means ±SEMs.

[c]N=17 mice/group (Veh), N=18 mice/group (SKF), N=14 mice/group (each dose of MLM).

[d]Behaviors were scored continually by photobeams or by the interval method beginning at 5 min after drug administration at 10-min intervals over 85 min.

[e]Welch ANOVA: drug [$F(3,27.609) = 18.851$, $p < 0.001$].

[ff]$p < 0.01$, [fff]$p < 0.001$, vehicle *vs*. SKF81297 and/or MLM55-38.
